# Green Tea Increases the Concentration of Total Mercury in the Blood of Rats following an Oral Fish Tissue Bolus

**DOI:** 10.1155/2015/320936

**Published:** 2015-08-02

**Authors:** Elsa M. Janle, Helene Freiser, Christopher Manganais, Tzu-Ying Chen, Bruce A. Craig, Charles R. Santerre

**Affiliations:** ^1^Department of Nutrition Science, Purdue University, 700 West State Street, West Lafayette, IN 47907-2059, USA; ^2^Department of Statistics, Purdue University, 250 North University Street, West Lafayette, IN 47907-2066, USA

## Abstract

Fish has many health benefits but is also the most common source of methylmercury. The bioavailability of methylmercury in fish may be affected by other meal components. In this study, the effect of green tea on the bioavailability of methylmercury from an oral bolus of fish muscle tissue was studied in rats and compared to a water treated control group and a group treated with meso-2,3-dimercaptosuccinic acid (DMSA), a compound used medically to chelate mercury. Rats were given a single oral dose of fish tissue via gavage and one of the treatments. Rats were given access to food for 3 h at 12 h intervals. They were dosed with each of the treatments with each meal. Blood samples were collected for 95 hours. Green tea significantly increased the concentration of total mercury in blood relative to the control, whereas DMSA significantly decreased it. In addition, feeding caused a slight increase in blood mercury for several meals following the initial dose.

## 1. Introduction

Mercury occurs naturally in the environment, but natural levels are greatly increased by human activity, including the combustion of fossil fuels [1–3], gold and silver mining, and the disposal of mercury containing products [4]. Elemental mercury accumulates in aquatic environments and is converted to methylmercury by microorganisms [5]. Methylmercury enters the human food chain primarily through fish. The highest concentrations of mercury occur in the large long-lived species [3] but high levels may also occur in fish living in highly polluted areas.

The health benefits of fish should ideally be balanced against the risks by consumption of low mercury fish. However, many consumers do not know or care about these distinctions and consume the fish they like. Therefore, it is important to understand the factors that might increase or decrease the bioavailability of the methylmercury in fish.

The bioavailability of methylmercury from fish is high with 90–95% being rapidly absorbed across the intestinal membrane [5, 6]. Once absorbed mercury binds to proteins [6], between 1 and 10% of the absorbed dose is found in the blood and 90% of the blood burden is in the red blood cells bound to the cysteine residues of hemoglobin [7]. Methylmercury is lipid soluble and is distributed to the fat rich tissues [5]. About 10% of the body burden of mercury is found in the brain [7]. It is also resecreted into the gastrointestinal tract through enterohepatic circulation [8]. It is possible that subsequent meals may promote reabsorption of this mercury resulting in slight postmeal spikes of plasma mercury.

Other factors which may affect bioavailability of methylmercury include the dietary components of a mixed meal [6] including dietary fibers and phytochemicals. Garlic contains potential chelating chemicals which can potentially increase the excretion of methylmercury [7].* In vitro* studies have shown that wheat bran decreases the bioaccessibility of methylmercury [8]. An* in vivo* study by Rowland et al. [9] showed that wheat bran but not pectin or cellulose can increase elimination of mercury and decrease brain concentrations.

Tea has been shown to affect the bioavailability of metals. It has been associated with iron deficiency in humans [10] and has been shown in a human clinical study to lower nonheme iron absorption [11]. One human clinical study [12] showed a decrease in iron, zinc, and magnesium with green tea treatment. It is therefore possible that green tea might decrease mercury absorption by a similar mechanism. In* in vitro* digestion studies by He and Wang [13], green tea has been shown to decrease the bioaccessibility of mercury from fish. Previous* in vitro* studies in our lab have demonstrated that both green and black tea decrease the bioaccessibility of methylmercury from fish [14]. These results lead to the hypothesis that green tea would also reduce the bioavailability of methylmercury* in vivo*.

The goal of this study was to investigate the effect of green tea on the bioavailability and toxicokinetics of mercury from an oral bolus of high-mercury fish tissue in rats and compare it with the effect of meso-2,3-dimercaptosuccinic acid (DMSA), a drug used in cases of mercury poisoning to eliminate mercury by chelation [15].

## 2. Materials and Methods

### 2.1. Materials

Meso-2,3-dimercaptosuccinic acid, ~98%, (DMSA) was obtained from Sigma Aldrich (St. Louis, MO).

Green tea extract (Nestle) was a gift of Mario Ferruzzi, Department of Food Science, Purdue University. The green tea polyphenol concentration in gallic acid equivalents (GAE) was 435 mg GAE/g green tea as analyzed by the Folin assay [16]. The catechin content of the green tea was determined by HPLC-ECD as described by Peters et al. [17]. Each gram of green tea contained 125 mg epigallocatechin, 30 mg epicatechin, 243 mg epigallocatechin gallate, and 35 mg epicatechin gallate.

Green tea and DMSA were analyzed for mercury contamination using the DMA-80 Mercury Analyzer (Milestone, Inc., Monroe, CT). Green tea contained 0.458 ± 0.032 ng/g and DMSA contained 5.361 ± 0.087 ng/g.

### 2.2. Animals

All animal procedures were approved by the Purdue Animal Care and Use Committee. Fifteen male Sprague-Dawley rats weighing 250–300 g were obtained from Harlan (Indianapolis, IN). They were placed on an AIN-93M polyphenol-free diet (Dyets, Bethlehem, PA) with water* ad lib* and allowed to acclimate for 6 days.

Fish tissue from a 658 kg sword fish was obtained from Santa Monica Seafood Company (Rancho Dominguez, CA). The fish tissue was analyzed for mercury content by thermal decomposition-amalgamation/atomic absorption spectrophotometry (TDA/AAS) [18, 19]. Fish tissue was ground in a food processor and 50 mg of tissue was placed in steel boats and analyzed in a DMA-80 Mercury Analyzer. Fish tissue contained 1.32 ± 0.01 mg mercury per kg fish tissue.

### 2.3. Experimental Plan

In order to train rats to eat when food was placed in the cage, the rats were placed on a restricted feeding schedule. The rats were given food* ad lib* for 3 hours. Then the food was removed and the rats were fasted for 9 hours. This schedule was repeated for 3 days. Water was provided* ad lib* at all times. An oral gavage tube was also inserted each day to familiarize rats with the procedure.

Rats were surgically implanted with a femoral vein catheter under isoflurane anesthesia. They were placed in a Culex automated* in vivo* sampling system (Bioanalytical Systems, West Lafayette, IN). Rats were allowed to recover from surgery for 48 hours. To maintain patency the Culex injects 15 *μ*L of dilute heparinized saline (20 U/mL) every 10 minutes.

Rats were divided into 3 treatment groups (*n* = 5): control (water), green tea extract (357 mg/kg), and DMSA (120 mg/kg). DMSA and green tea extract were dissolved in 0.5 mL of water. The control treatment was 0.5 mL of water. A baseline blood sample (5 *μ*L) was drawn. A slurry was made by grinding the fish tissue in a food processor. Rats were dosed with the 4 g fish tissue slurry/kg body weight plus treatment, by oral gavage. This was equivalent to a dose of 5.24 *μ*g mercury/kg body weight. The rats were dosed with the fish tissue only once at the beginning of the study. Rats were dosed with treatment at the start of each feeding period. Blood samples (5 *μ*L) were taken every hour for the first 8 hours, then every 2 hours until 80 hours after dose, and finally every 3 hours until 95 hours after dose. At the conclusion of the study, rats were terminated with carbon dioxide overdose.

### 2.4. Sample Analysis

Blood samples were analyzed for total mercury with the TDA/AAS by the method of Stube et al. [20].

### 2.5. Data Analysis

Blood mercury concentrations are expressed as mean ± SEM. AUC was calculated by the trapezoidal method. *T*_1/2_ and *C*_elim_ were calculated using Excel spreadsheet pharmacokinetic function addins developed by Usansky et al. [21].

Data were analyzed using SAS statistical software package version 9.3 (Cary, NC). The pharmacokinetics data were analyzed by the GLM procedure for repeated measures with* post hoc* Tukey analysis of differences at various time points. Area under the curve (AUC), maximum concentration (*C*_max⁡_), time of maximum concentration (*T*_max⁡_), elimination rate constant (*C*_elim_), and the half-life (*T*_1/2_) were analyzed by one-way ANOVA. Pairwise treatment differences were analyzed using the Tukey Studentized Range test. Tukey analyses were done to compare differences between treatments. Differences were considered significant at *P* < 0.05.

To compare transient postprandial elevations in plasma mercury levels over time across treatments, a linear mixed model framework (PROC MIXED) was used to describe difference in plasma mercury over time across individuals and treatments. For each treatment, a 6th-order polynomial was fit (i.e., treatment-specific coefficients) with the individual rat curves varying about them (first 3 polynomial coefficients were considered random). Additionally, indicator variables were used to denote whether or not a rat was initially fed that hour. Likelihood ratio tests were used to assess whether these indicator variables explained a significant amount of variation and if so, whether there were differences in spikes across treatments and feeding episodes.

## 3. Results

**Figure 1:**
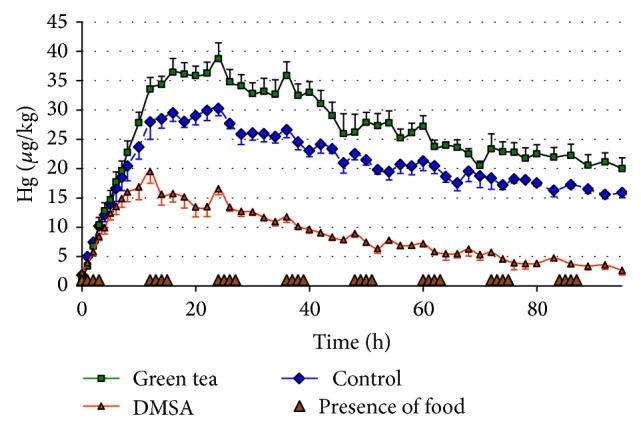
Blood mercury concentration after oral gavage of high mercury fish tissue with green tea, DMSA versus control.

**Figure 2:**
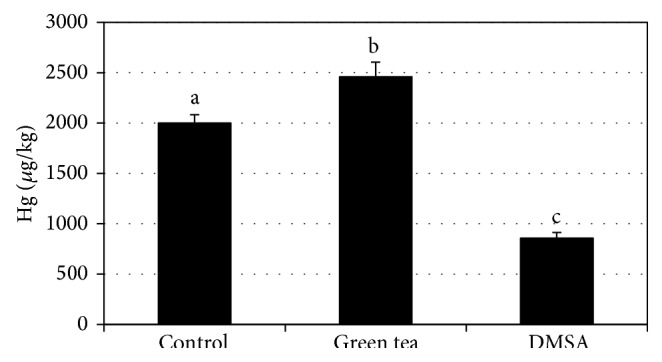
Area under the curve for mercury concentration versus time for time = 0 to 95 h. The area under the curve was significantly different for each group (*P* < 0.05).

The total blood mercury levels of over 96 hours after dose are illustrated in Figure 1. The baseline blood mercury concentrations for the three groups were the following: control (1.8 ± 0.6 *μ*g/kg), green tea (1.3 ± 0.2 *μ*g/kg), and DMSA (2.3 ± 0.5 *μ*g/kg). The area under the curve for the three groups from 0 to 95 h is shown in Figure 2. The AUC was significantly different for all groups. The AUC for DMSA treatment (857 ± 56 *μ*g h/kg) was significantly lower than the AUC of the control group (2000 ± 83 *μ*g h/kg). Green tea increased the AUC significantly (2460 ± 145 *μ*g h/kg) compared to the control group. The pharmacokinetic parameters are shown in Table 1. The maximum concentration (*C*_max⁡_) of the tea treated group (37.49 ± 2.48 *μ*g/kg) is not significantly different from the control group (31.21 ± 1.50 *μ*g/kg). The *C*_max⁡_ for the DMSA treated group (18.64 ± 2.00 *μ*g/kg) is significantly lower than the *C*_max⁡_ of the control group and green tea treated group. The time of maximum concentration (*T*_max⁡_) was not significantly different between the tea treated group (22.33 ± 1.96 h) and the control group (20.33 ± 1.20 h). The *T*_max⁡_ for the DMSA treated group (12.00 ± 0.00 h) was significantly shorter than the green tea or control group. The *C*_elim_ was not significantly different for the control (0.0076 ± 0.0007) and green tea (0.0080 ± 0.0008) groups. The *C*_elim_ for the DMSA group (0.0241 ± 0.0046) was significantly higher than both the control and green tea groups. The blood mercury concentrations were not different for the three treatments for the first 8 hours. At 10 hours, the mercury concentration of the DMSA group was significantly lower (*P* < 0.05) than the control or green tea groups and remained significantly lower for the rest of the study. At 14 hours there was a trend toward a higher blood mercury in the green tea group than in the control group (*P* = 0.08), and by 18 hours the blood mercury in the green tea group was significantly higher (*P* < 0.05) than the control group. For all except 5 time points (70, 76, 78, 89, and 90 hours) the mercury of the green tea group remained significantly higher. For 3 of the time points that did not reach significant differences, 0.05 < *P* < 0.1.

Including treatment- and time-specific effects of a meal was highly significant (Chisq = 104.9, df = 24, and *P* < 0.0001). Due to the limited sample size, treatment differences were not detected but positive spikes were significant at hours 12, 24, and 36. While these spikes were not found significantly different, the largest spike was at 12 h, followed by 24 h, and then 36 h.

## 4. Discussion

From our previous studies which demonstrated that green tea decreased the bioaccessibility of mercury from fish [14], it was reasonable to hypothesize that green tea would reduce mercury bioavailability* in vivo*. Other* in vitro* digestion studies [13, 22] have also shown that green tea significantly decreased the bioaccessibility of mercury from fish tissue. Also the polyphenols present in tea are known to chelate metals [23, 24]. Previous studies have shown that green tea can affect the body's mineral status. In a human study designed to study the effect of green tea on obesity it was found that 3 months of green tea supplementation significantly decrease serum iron levels and significantly increased magnesium and zinc status [12]. Green tea was also shown to have a high absorptive capacity for heavy metals [25]. Other nutritional factors such as wheat bran have been shown to decrease the absorption of methylmercury [9].

The rat has been validated as a model for mercury toxicokinetic studies [26]. The objective of this study was to investigate the potential of green tea to reduce the bioavailability of methylmercury from a fish tissue meal using the rat model and compare it to the standard medical treatment for mercury chelation, DMSA. Contrary to our hypothesis that green tea would reduce the bioavailability of mercury from fish, it significantly increased it. The AUC for mercury concentration x time was significantly greater for green tea treated rats than for controls. The *C*_max⁡_ was also greater for the green tea treated group but the difference was not significant. The result was not due to the small amount of mercury in the tea. The mercury in the fish dose was 30,000 times greater than the mercury in the tea. The DMSA treatment resulted in a significant decrease in methylmercury consistent with other studies [15]. Canuel et al. [27] also found in a 3-day human study that consumption of tea with a fish meal resulted in 40% higher blood mercury levels than consumption of fish meals without tea. Other discrepancies between* in vitro* and* in vivo* bioaccessibility and bioavailability have been noted. Vázquez et al. [28] used Caco-2 cells to investigate intestinal absorption of methylmercury and found only moderate absorption in contrast to the high absorption found* in vivo*. The* in vitro* model used to determine bioaccessibility [14] does not have a large intestine component and therefore cannot account for enterohepatic circulation. It also cannot account for effects of metabolism of flavonoids by microbiota which might release mercury from flavonoid interaction. The actual mechanism for the increased bioavailability of methylmercury with green tea is not known and requires further investigation. Canuel et al. [27] postulated that tea increased mercury due to enterohepatic circulation and increased release of Hg from liver. Methylmercury complexes with glutathione in the liver [29] and is secreted into the bile. Green tea has been shown to increase glutathione levels [30]. This could result in an increased delivery of the mercury from liver stores to the intestine for reabsorption. The difference in blood concentrations between control and green tea treatment is not seen until 10 hours and does not become significant until 18 hours. Therefore initially there does not seem to be any difference in absorption between the different treatments. It is possible that the increased blood level of methylmercury with green tea treatment occurs only after there is a green tea induced increase in glutathione and an increased secretion of the liver methylmercury-glutathione complex into the bile which can be delivered to the intestine for reabsorption.

The *T*_1/2_ was also not significantly different between controls and the green tea treated group but was significantly decreased by DMSA treatment. The *T*_1/2_ for mercury in blood was about 90 h in untreated rats. Estimates of mercury half-life in humans vary with chemical form, level, and duration of mercury exposure. Elemental mercury in the blood pool has a rapid half-life of 1 to 3 days followed by a slower decline with a half-life of 1–3 weeks [31], whereas the half-life of methylmercury is about 50 days [32]. Long industrial exposures, which build up tissue pools, can result in half-lives that range from 40 to 90 days [33]. Half-lives in different tissues may differ from blood. There is evidence that the half-life in brain may be considerably longer than in blood. Burbacher et al. [34] found that in infant monkeys the half-life of methylmercury in blood was 19 days and in brain was 60 days.

In this study we observed slight but significant increases in plasma mercury following meals in all treatment groups. These responses are seen in all treatment groups but are seen most clearly in the green tea and DMSA groups. The meal responses appear to diminish with time. The mechanism of these meal responses is not known but may be related to the enterohepatic circulation of mercury. Methylmercury is taken up by the liver and secreted into bile complexed with glutathione and released into the intestine [29] where it can be reabsorbed. Tsutomu et al. [35] demonstrated in an isolated rat intestine that methylmercury is also absorbed from the intestine complexed with cysteine and cysteine-glycine. These complexes are also formed in bile. A meal would increase the flow of bile which would release the mercury load in the intestine where it could be reabsorbed and may be responsible for the slight increase in plasma mercury seen following meals. This is supported by the fact that, in studies in rats where the bile duct was ligated, mercury absorption was decreased [8, 36]. Also one or more of the components of the meal may also promote the reabsorption of mercury [6]. It has been suggested by Bridges and Zalups [8] that amino acids and peptides from food form complexes with mercury and are absorbed by amino acid or peptide transporters.

## 5. Conclusion

Green tea increases the concentration of total mercury in rat blood following dietary intake of fish. Meals increase slightly the blood concentration of mercury in control, green tea, or DMSA treated rats that have consumed mercury containing fish.

## Figures and Tables

**Table 1:** Plasma pharmacokinetic parameters from Sprague-Dawley rats gavaged with high mercury fish slurry and treated with water, green tea, or DMSA^a,b,c^.

	*C*_max⁡_ (*µ*g/kg)	*T*_max⁡_ (h)	*C*_elim_ (h^−1^)	*T*_1/2_ (h)
Control (water)	31.21 ± 1.50^a^	20.33 ± 1.20^a^	0.0076 ± 0.0007^a^	93.8 ± 7.0^a^
Green tea	37.49 ± 2.48^a^	22.33 ± 1.96^a^	0.0080 ± 0.0008^a^	90.9 ± 8.3^a^
DMSA	18.64 ± 2.00^b^	12.00 ± 0.00^b^	0.0241 ± 0.0046^b^	33.2 ± 5.0^b^

^a^ *C*_max⁡_ = maximum plasma mercury concentration; *T*_max⁡_ = time of maximum plasma mercury concentration; *C*_elim_ = elimination rate constant; ^b^data are expressed as mean ± SEM.

^c^Different letters indicate a significant (*P* < 0.05) difference in plasma pharmacokinetic parameters between different treatments.
